# The MBCRC Advocate Researcher Program (MARP): connecting advocates and researchers as collaborative partners in cancer research

**DOI:** 10.1038/s41523-025-00771-6

**Published:** 2025-06-21

**Authors:** Hillary S. Andrews, Igor L. Bado, Amy Beumer, Isaac S. Chan, Janice Cowden, Debbie Denardi, Gloria V. Echeverria, Brooke L. Gates, Marybeth Gilliam, Christine Hodgdon, Adrian V. Lee, Joan Mancuso, Julia Maues, Steffi Oesterreich, Michael Papanicolaou, Katherine E. Pendleton, Bob Riter, Kelly Shanahan, Anh M. Tran-Huynh, Pavitra Viswanath, Stephanie Walker, Alana L. Welm, Michelle M. Williams, Garhett L. Wyatt, Josh Newby

**Affiliations:** 1 Friends of Cancer Research, Washington, DC USA; 2 Icahn School of Medicine at Mount Sinai, New York, NY USA; 3 METAvivor and Project Life, Mason, OH USA; 4 University of Texas Southwestern Medical Center, Dallas, TX USA; 5 Independent Patient Advocate, Bradenton, FL USA; 6 Independent Patient Advocate, North Miami Beach, FL USA; 7 Baylor College of Medicine, Houston, TX USA; 8 University of Utah Huntsman Cancer Institute, Salt Lake City, UT USA; 9 Outperform Cancer, Charlotte, VT USA; 10 GRASP, Baltimore, MD USA; 11 University of Pittsburgh, Pittsburgh, PA USA; 12 Independent Breast Cancer Advocate, Philadelphia, PA USA; 13 GRASP, Washington, DC USA; 14 Albert Einstein College of Medicine, New York, NY USA; 15 Independent Patient Advocate, Ithaca, NY USA; 16 METAvivor, Lake Tahoe, CA USA; 17 Independent Patient Advocate, Tarboro, NC USA; 18 Texas A&M University, College Station, TX USA; 19 Theresa’s Research Foundation, Houston, TX USA

**Keywords:** Breast cancer, Metastasis, Preclinical research, Translational research

## Abstract

Involving patient advocates as partners in cancer research improves research and provides favorable experiences for both the researcher and the advocate. Previous work demonstrates challenges to establishing relationships between researchers and advocates, including uncertainty about why the relationships are necessary, how to establish them, what to say, and how they should be structured. To overcome these challenges, we established the Metastatic Breast Cancer Research Conference (MBCRC) Advocate Researcher Program (MARP) at the MBCRC in 2023. We outline the approach to the program to serve as a model for others interested in performing similar activities and report findings from surveys to establish evidence about the value of these relationships. The program connected 21 pairs of researchers and advocates, and participants responded to surveys about their experience, largely describing positive outcomes. Our hope is that a program like this could be used at any cancer conference in the future as we continue to encourage advocates and researchers to work together.

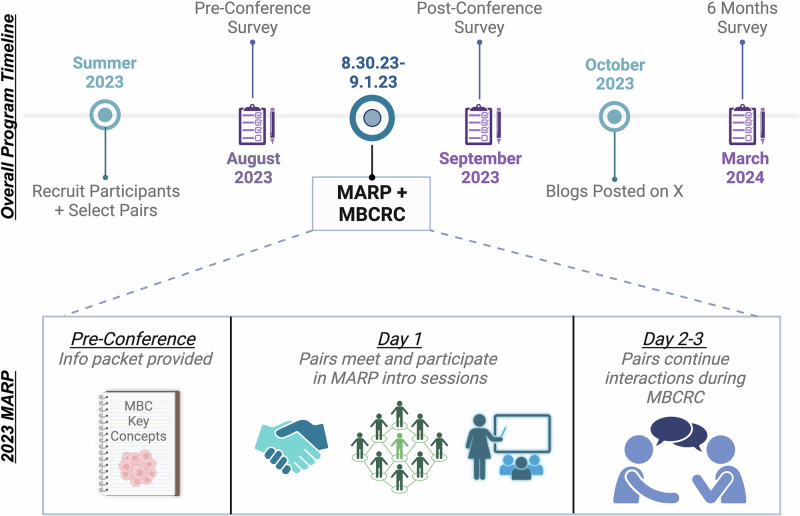

## Introduction

Involving patient advocates as partners in cancer research from project development through the dissemination of results improves research and provides favorable experiences for both the researcher and the advocate^1–4^. Some incredible initiatives include advocates at conferences such as the ABC Global Alliance, the Alamo Breast Cancer Foundation at the San Antonio Breast Cancer Symposium, and the Scientist ←→ Survivor Program through AACR^5–7^, but these programs do not necessarily focus on establishing long-term partnerships. We previously identified major challenges for establishing relationships between advocates and researchers^8^: (1) It is not always clear why research advocacy is necessary; (2) both researchers and advocates worry about saying the wrong thing; (3) they do not know how to begin working with each other, including how to find one another; and (4) they do not know how the relationship should be structured, including what role each would play when working together and how to ensure advocates receive appropriate remuneration. To overcome these challenges, we established a formal program to connect researchers and advocates and determine the merits, challenges, and limitations.

The first iteration of the program took place at the Metastatic Breast Cancer Research Conference (MBCRC) in 2023, a conference that prioritizes including advocates, but did not previously have an established framework for forming partnerships. We called the program the MBCRC Advocate Researcher Program (MARP) and hypothesized that a more formal program that explicitly targeted the above challenges would enable bidirectional relationships between advocates and researchers. Herein, we describe our approach, the experiences of the participants, and opportunities to organize future programs.

## Results

### Participants

**Fig. 1:**
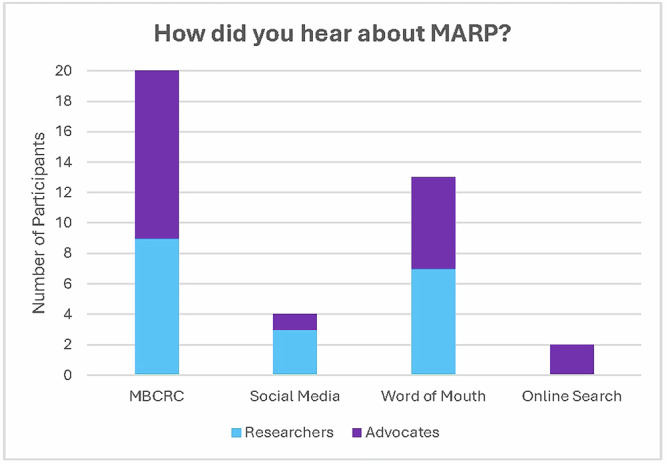
The majority of participants heard about MARP through previous MBCRC-related emails. Researchers and advocates respond when asked how they heard about the program. Since the survey question was an open text box, we reviewed the results and created categories based on responses.

In total, 21 researchers and 21 advocates applied for the program, and all were ultimately accepted, creating 21 pairs. Of the 21 researchers, 19 filled out the pre-conference survey, showing there were 9 graduate students, 6 postdoctoral fellows, and 4 junior faculty members who were paired with advocates. The majority had previously worked with advocates (15/19, 79%). Of the 21 advocates, 20 filled out the pre-conference survey and all (20/20, 100%) had previous science or advocacy training experience. When asked how they heard about the program, most participants heard through MBCRC-related emails (Fig. 1). More researchers than advocates learned about the program from social media.

### Pre-conference survey results

When asked about preparing for the training, about half of the researchers (10/19, 53%) and three-quarters of the advocates (15/20, 75%) said they prepared by reading research articles and reaching out to other researchers who work with advocates. The majority of people in both groups had read about or discussed the value of working with each other (13/19, 68% of researchers and 12/20, 60% of advocates). From the researcher’s perspective, in open survey responses, advocates add important perspective to research directions and priorities, including ensuring the research not only impacts survival but also ensures patients receive quality care (e.g., considering potential side effects of novel treatments). Additionally, advocates remind researchers why they do cancer research, provide motivation to continue working in the lab, and center their research ideas around patient needs. Further, working with advocates requires researchers to describe complex topics in more lay terms. From the advocate’s perspective, working with researchers helps them increase their own knowledge that they then share with others, including informing peers about clinical trial opportunities and sharing the value of engaging in research. They can share their experiences as a patient, including their treatment and clinical experience, ultimately leading to improvements in research, which is rewarding. Both groups appreciated the value of joint exploration and collaboration.

When asked how they felt about the program, more researchers mentioned being nervous (5/19, 26%) than advocates (2/20, 10%). When describing their feelings about the program, 15 advocates and 13 researchers mentioned the term “excitement.” From both groups, the comments reflected a blend of excitement, anticipation, and a touch of nervousness, with a shared commitment to learning, partnering, and building meaningful connections. Many researchers mentioned this was their first one-on-one in-person interaction with patient advocates, which added a layer of nervousness but also a high level of anticipation.

### Post-conference survey results

**Fig. 2:**
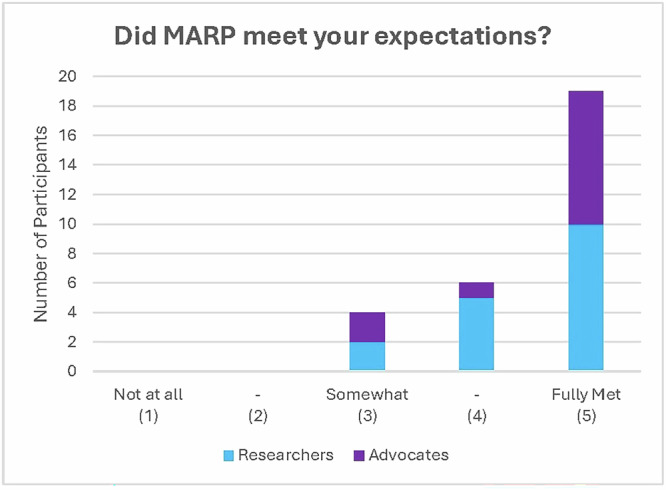
The majority of participants felt that MARP fully met their expectations. Participants’ responses to the question “Did MARP meet your expectations?” (1 = not at all, 3 = somewhat, 5 = fully met).

All participants were asked about their experience with MARP in a survey sent 1 week after the conference ended. There were 17 researchers (out of 21; 81%) and 12 advocates (out of 21; 57%) who filled out the post-conference survey. When asked to score whether the program met their expectations, the majority of participants said their expectations were fully met (Fig. 2).

Most participants reported feeling prepared due to pre-conference resources and their background; however, some faced challenges related to partner interactions. On the first day of the conference, name tags were placed for the participants to sit near their partners. With so many pairs meeting for the first time, some of the pairs missed each other initially, including one pair who was never able to connect (leading to 20 pairs participating in the program). In surveys completed after the conference, participants noted they would have appreciated being introduced to their partner via email to allow for a virtual meeting ahead of the conference.

When asked about goals they set for the following six months, the participants mentioned monthly communication, lab meeting attendance, lab tours, and seeing each other at conferences. The researchers hoped to understand patient obstacles and learn about patient priorities. Advocates were interested in understanding research projects and helping review presentations and grants to ensure the language was understandable for broader audiences. Some participants mentioned they did not identify goals or struggled to know what goals to set, aligning with previous findings that even if these partnerships can be established, people do not always know what the relationships should look like. One advocate even mentioned this in their survey, stating, “More guidance during the conference on how the pairs could work together would be nice.”

While the majority of participants described a positive experience with MARP, many provided suggestions for how to improve the program moving forward. One researcher requested additional expectation setting, including “there could have been more structure, clearer ‘rules’ and defined expectations about the MARP for it to work better.” Two researchers asked for longer orientation, with one also suggesting a formal debriefing session at the end of the conference. One researcher suggested that the advocates select their researcher based on their research and interests, rather than having the organizers choose.

### Blogs

All 20 pairs who successfully matched completed their blog posts, which were subsequently posted on the Theresa’s Research Foundation website (available from: https://theresasresearch.org/marp). During the month of October, the blogs were shared via X on each weekday. The blogs highlighted participant experiences with the program and the value of working together.

### 6-Month survey

Six months after the conference, a third survey was sent to all participants, and 14 researchers and 15 advocates filled it out. Only 50% (7/14) of researchers and 40% (6/15) of advocates said they maintained regular contact with their MARP partner. Busy schedules and geographic distance were factors that affected contact for some pairs. Those who did maintain contact did so through regular Zoom calls, text and email communication, manuscript reviews, conference presentations (posters/talks), and working on grants together. When asked whether further support or training would improve the outcome of MARP, both researchers and advocates suggested additional structured opportunities for connecting virtually. Some mentioned they simply forgot about the program, and the 6-month survey email reminded them, suggesting more regular reminders might support improved, longer-term connections. One researcher suggested it would be helpful to have a more detailed outline of activities partners can do together.

When asked about the value of the program, many researchers appreciated how the advocate perspective influenced their research, including more effective ways to communicate scientific results and ways to link their research questions to improving patient outcomes. One researcher said, “When we looked at the data, as a researcher, I immediately looked for any significant changes in a gene expression level. However, my advocate partner was looking for the relationship between the expression level and patient survival data. That was a very educational moment that I should think more about the patient outcomes.”

## Discussion

To overcome challenges with researchers and advocates working together, we established a program and initiated the first iteration at the MBCRC in 2023. The program connected 21 pairs of researchers and advocates, and participants responded to surveys about their experience, largely describing positive outcomes. Some mentioned challenges with establishing lasting relationships. We addressed the four previously identified challenges for establishing relationships between advocates and researchers: (1) It is not always clear why research advocacy is necessary; (2) both researchers and advocates worry about saying the wrong thing; (3) they do not know how to begin working with each other, including how to find one another; and (4) they do not know how the relationship should be structured, including what role each would play when working together and how to ensure advocates receive appropriate remuneration. The partnership also sets the stage for future collaboration.

The program demonstrated the value of relationships between researchers and advocates. Many participants noted that they were aware of advocates and researchers working together before participating in the program and thus shared some of the perceived value. Working together established their own personal experiences, which were then shared via blog posts. Additionally, survey responses demonstrated that the researchers felt their research improved because it became more patient-centric, while advocates appreciated their voice being heard and learning about research that they were able to share with their community.

One potential limitation of this work is that the participant pool was biased by the applicants probably already being interested/intrigued by these partnerships. Because many of the participants were already aware of the value of these relationships, we missed perspectives from people who had never worked together or previously did not believe there was value. Future iterations of the program will attempt to identify participants who have never worked together or heard of these types of relationships. In the future, it may be beneficial to introduce the program to locations where patients are being treated. A challenge will be to get people on board if they do not see the value, however, a financial incentive for having conference fees covered may be helpful.

The program provided a safe space for advocates and researchers to learn from one another. During the orientation, we discussed the importance of being open and honest as experiences were shared, which included recognizing that partners were being vulnerable and may say the wrong thing. To make initial conversations even more fruitful and focused on research, it would be helpful to introduce partners via email before the conference, so they have a chance to meet via a video conference ahead of time. That way, initial discussions can take place, and the conference can be a time for growth. Additionally, a smaller number of pairs (i.e., 5–10 pairs) may help them to not only learn from one another, but also learn from the other participants, which was challenging because there were so many participants.

The program helped overcome the challenge of being unsure of how to establish a working partnership between advocates and researchers. However, it was not always clear where the partnership could go long-term. Many pairs mentioned they struggled identifying goals for the future of how their partnership should continue. A session describing approaches to continued connections, either at the end of the conference or virtually after the conference, would be a great way to share best practices and set the stage for success.

While the surveys and blog posts demonstrated qualitative results and anecdotal evidence demonstrating the value of advocates and researchers working together, more quantitative measurements of success would be helpful. For example, understanding grant success or the number of manuscripts accepted (including impact factors) of those who work with advocates compared with those who do not may further demonstrate the value. Additionally, more explicit examples of how research directions changed may encourage researchers to work with advocates.

Advocate/researcher relationships are important to ensure research is patient-focused and improves outcomes. The benefit of hosting MARP during a conference is that it provides the researchers and advocates with plenty of material to discuss during breaks, at the poster session, and after the conference. Researchers were able to share the work in a way that is understandable to broader audiences, which helps them digest it more effectively, and the advocates can ask the researchers questions that help to better understand the work that is being presented. Our hope is that a program like this could be used at any cancer conference in the future as we continue to encourage advocates and researchers to work together.

## Methods

### Participant selection and expectations

This study was performed in accordance with the Declaration of Helsinki and received ethical approval from Baylor College of Medicine. Informed consent to participate in the program was obtained from all participants. We focused on advocates and cancer researchers from academic institutions (with a preference for early career investigators, including PhD students, postdoctoral fellows, and junior faculty). Advocates are participants who have been diagnosed with breast cancer at any time or their caregivers and engaged in advocacy activities, including but not limited to research advocacy. A research advocate is a patient or advocate who uses their own personal experiences, along with insights from the broader patient community, to inform scientific researchers and research processes. As the conference goals focused on metastatic breast cancer research, we prioritized researcher applicants who focus their work on metastatic and/or breast cancer research. For researchers, our goal was to include early career investigators who would benefit from establishing these relationships in the formative years of their careers to serve as a foundation for supporting their research goals.

To identify interested participants, we shared a call for applicants via social media (including X and LinkedIn), sent emails to previous MBCRC attendees, posted on the MBCRC website to share information about the program and solicit applications, and mobilized research advocacy champions—including partner organizations—to promote the program within their networks. To be considered for the program, researcher applicants provided their CV and background information about their research, while advocate applicants provided their resume and information about their experience with cancer. Participants were selected in a way that balanced the cohort with respect to expertise, background, experience with cancer, and goals. Partnerships were created by program organizers, who attempted to align research interests with patient experiences and similar geographic locations.

MARP participants were expected to attend MBCRC in person in Park City, Utah. Accepted participants had their conference registration and travel fees covered. The MBCRC was a two-and-a-half-day conference that occurred from Wednesday, August 30–Friday September 1, 2023. Accepted participants filled out three surveys: (1) before the conference began (pre-conference survey), (2) in the weeks following the conference (post-conference survey), and (3) 6 months after the conference (6-month survey).

The participants were required to attend a pre-conference training on the first day of the conference (see below for details), which was an opportunity for each researcher-advocate pair to get to know each other by introducing themselves and discussing what each wanted to contribute to and learn from the other. The pairs were encouraged to meet with their partner 1–2 times during MBCRC. After the conference, the MARP participants wrote blog posts with their partners that were posted on the Theresa’s Research Foundation website and shared via social media (including X and LinkedIn) during Breast Cancer Awareness Month in October 2023. Participants were encouraged to continue working with their partners after the conference. Program participants consented to the publication of this paper, many of whom are included as authors on this work.

### MARP at MBCRC

Before the conference, participants accepted into the program were provided with background information about cancer research and examples of patient advocates and researchers working together^4,8^. We created a reference document with researcher and advocate input that outlined the key terms and concepts related to metastatic breast cancer (MBC; available from https://theresasresearch.org/advocacy-program).

On the first day of the conference, we hosted a 4-h opening session to provide a strong foundation and contextualization of MBC research for researchers and advocates attending MBCRC. The first hour was an orientation for MARP participants to review expectations of the program and introduce the pairs for initial discussions. To stimulate discussion, we established a question/prompt handout that included questions “for advocates” and “for researchers” (see Supplementary Materials).

During the next 3 h of the orientation, all conference attendees were invited to attend. A patient advocate/researcher pair who had been working together for over 2 years presented approaches for interpreting data. Next, a PhD student presented the biology of metastasis and the models used to do research. Finally, another established patient advocate/ researcher pair presented on the history of breast cancer research and advocacy.

### Blogs

Following the conference, pairs were asked to create a ~500–1000-word blog post. The topic was determined by the pairs, but the goal was to show the value of the relationships and the lessons they have learned from each other. Blogs were posted on Theresa’s Research Foundation website (available from: https://theresasresearch.org/marp) and shared via X during October 2023 to align with Breast Cancer Awareness Month.

### Surveys

We created surveys with the goal of understanding the success of the program and providing concrete examples of the value of researcher-advocate partnerships. Participants received three surveys: one that they were asked to complete before the conference began (pre-conference survey), one week after the conference ended (post-conference survey), and one 6 months after the conference (6-month survey). Surveys for the advocates and researchers were slightly different, and questions are included in Table 1. Survey findings were compiled and reviewed by the authors.

**Table 1:** Survey Questions

	Advocates	Researchers
Pre-conference survey	• How did you hear about MARP? (open response) • Do you have previous science or advocacy training/experience? (Y/N) • Did you do any preparation for this training (reading research articles, outreach to other advocates, etc.) (Y/N) • Have you read/discussed the value of working with a researcher? (Y/N) If yes, how do you perceive the value? (open response) • What was your interest in applying for the program? (open response) • How are you feeling about interacting with your researcher? (open response) • What are your expectations for this program? (open response) • Additional comments (open response)	• How did you hear about MARP? (open response) • Do you have previous experience working with advocates? (Y/N) If yes, please describe how you have worked with advocates (open response) • Did you do any preparation for this training (reading research articles, reaching out to other researchers who work with advocates, etc.) (Y/N) • Have you read/discussed the value of working with an advocate? (Y/N) If yes, how do you perceive the value? (open response) • What was your interest in applying for the program? (open response) • How are you feeling about interacting with your patient partner? (open response) • What are your expectations for this program? (open response) • Additional comments (open response)
Post-conference survey	• Did the MARP program meet your expectations? (scale) • How much did you interact with your MARP partner during the event? (scale) • Did you feel prepared/supported to discuss research presentations or concepts with your MARP partner? (Y/N) • What aspect of the MARP training was most helpful for your experience of the conference and future interactions with researchers? (open ended) • What goal will you set for yourself that you wish to achieve in 6 months related to your experience with MARP? (open response) • Have you made plans to stay in contact with your MARP partner? (Y/N) If yes, what are your plans (open response) • Additional Comments (open response)	• Did the MARP program meet your expectations? (scale) • How much did you interact with your MARP partner during the event? (scale) • Did you feel prepared/supported to discuss research presentations or concepts with your MARP partner? (Y/N) • What aspect of the MARP training was most helpful for your experience of the conference and future interactions with advocates? (open ended) • What goal will you set for yourself that you wish to achieve in 6 months related to your experience with MARP? (open response) • Have you made plans to stay in contact with your MARP partner? (Y/N) If yes, what are your plans (open response) • Additional Comments (open response)
6-Months survey	• Have you maintained regular contact with your MARP partner? (y/n) • If yes, how have you stayed in contact? If no, is there a reason why? (open response) • What was the goal you set for you and your MARP partner that you wished to achieve in 6 months related to your experience with MARP? (Open response) • Did you achieve this goal? If yes, how? If no, what could have helped you to do so? (open response) • Is there further support or training that would improve the outcome of MARP? (Open response) • Do you feel like there was value in participating in MARP? (y/n) • If yes, what was the value? If no, what would have improved your experience? (Open response) • Overall, how satisfied are you with MARP? (Select one: Very satisfied, somewhat satisfied, neither satisfied nor dissatisfied, somewhat dissatisfied, very dissatisfied) • Additional Comments (open response)	• Have you maintained regular contact with your MARP partner? (y/n) • If yes, how have you stayed in contact? If no, is there a reason why? (open response) • What was the goal you set for you and your MARP partner that you wished to achieve in 6 months related to your experience with MARP? (Open response) • Did you achieve this goal? If yes, how? If no, what could have helped you to do so? (open response) • Is there further support or training that would improve the outcome of MARP? (Open response) • Do you feel like there was value in participating in MARP? (y/n) • If yes, what was the value? If no, what would have improved your experience? (Open response) • Overall, how satisfied are you with MARP? (Select one: Very satisfied, somewhat satisfied, neither satisfied nor dissatisfied, somewhat dissatisfied, very dissatisfied) • Additional Comments (open response)

After all surveys were submitted, the authors reviewed survey responses and aligned on takeaways. For questions that were more quantitative and used numbers, values were averaged across all responses.

## Supplementary information

MARP Supplementary Materials

## Data Availability

The datasets generated and/or analyzed during the current study are not publicly available due to participant consent, but are available from the corresponding author on reasonable request. All other data are provided within the paper or Supplementary Information Files.
