# Supplementary material for: The MBCRC Advocate Researcher Program (MARP): connecting advocates and researchers as collaborative partners in cancer research
Source: NPJ Breast Cancer. 2025 Jun 21;11:60. doi: 10.1038/s41523-025-00771-6 (PMC12182560; doi:10.1038/s41523-025-00771-6)
Supplement: Supplementary file 1 — MARP Supplementary Materials [file 41523_2025_771_MOESM1_ESM.pdf]

The MBCRC Advocate Researcher Program (MARP): Connecting Advocates and Researchers as Collaborative Partners in Cancer Research

Supplementary Materials

MARP PARTNER DISCUSSION QUESTIONS

Ask an advocate

1. What is your cancer story?
2. What challenges did you face during the diagnosis?
3. What advocacy activities do you participate in? How did you get started in them?
4. What would you like a researcher to know about having your disease?
5. What is important to you when it comes to discoveries about your disease (e.g., a new drug for your disease)?

Ask a researcher

1. What kind of research do you do? Explain your project.
2. Why did you go into this area of research?
3. How do you think what you are studying will help a real live patient?
4. How do you determine your research priorities?
5. How and where do you get your funding?
6. How long do you think it will be before your research either saves lives, extends lives or improves the quality of cancer patients lives?

Discuss together

1. How might patient advocates work with researchers as integral part of a research team?
2. How does the researcher communicate about their work with people outside of your field? (Brainstorm how advocates and trainees can work together to accomplish this!)
3. Do you take time out occasionally to reflect on bigger picture issues and make occasional cross disciplinary connections in cancer research? Sometimes talking to a colleague working in another area can ask the most basic questions or stimulate other ideas in your work!
